# Quadriceps strength, patellar tendon quality, relative load exposure, and knee symptoms in male athletes before the anterior cruciate ligament reconstruction

**DOI:** 10.3389/fresc.2023.1283635

**Published:** 2023-10-19

**Authors:** Carla S. Pereira, Jasenko Klauznicer, Dustin Maree, Sean McAuliffe, Abdulaziz Farooq, Rod Whiteley, Taija Finni

**Affiliations:** ^1^ Rehabilitation Department, ASPETAR, Orthopaedic and Sports Medicine Hospital, FIFA Medical Centre of Excellence, Doha, Qatar; ^2^ Neuromuscular Research Center, Biology of Physical Activity, Faculty of Sport and Health Sciences, University of Jyväskylä, Jyväskylä, Finland; ^3^ Department of Physical Therapy & Rehabilitation Science, College of Health Sciences, Qatar University, Doha, Qatar

**Keywords:** ACL, knee, quadriceps, isokinetic, ultrasound tissue characterization, patellar tendon, relative load, tendon quality

## Abstract

**Introduction:**

Anterior cruciate ligament (ACL) injuries cause knee instability, knee pain, weight-bearing adjustments, and functional deficits but their association to patellar tendon quality is unknown. Our purpose was to investigate quadriceps strength, patellar tendon quality, relative load exposure, perceived knee stability, knee pain, extension angle, and time from ACL injury; in addition to examining their relative associations.

**Methods:**

Injured and uninjured legs of 81 male athletes of different sports with a unilateral ACL injury (18–45 years) were examined. Participants reported location and intensity of knee pain and their perceived stability using a numerical rating scale (NRS 0–10). Strength was tested with an isokinetic device. Tendon quality was measured using ultrasound tissue characterization. Means ± standard deviation (SD) of perceived knee stability, knee extension angle, knee pain, isokinetic quadriceps strength in relation to body mass, proportion of echo-types (I–IV), tendon volume, and number of days from ACL injury to assessment are reported. Values of effect sizes (ES) and correlations (*r_s_*) were calculated.

**Results:**

ACL injured leg demonstrated reduced reported knee stability (6.3 ± 2.5), decreased knee extension angle (−0.7 ± 3.1° vs. −2.7 ± 2.2°; ES = 0.7; *P *< 0.001), greater knee pain (NRS 3.1 ± 2.2 vs. 0.0 ± 0.1; ES = 2.0; *P *< 0.001), and 22% lower quadriceps strength (228.0 ± 65.0 vs. 291.2 ± 52.9 Nm/kg: ES = 1.2; *P *< 0.001) as compared to the uninjured leg. However, patellar tendons in both legs displayed similar quality. Quadriceps strength was associated with stability (*r_s_*_ _= −0.54; *P *< 0.001), pain (*r_s_*_ _= −0.47; *P *< 0.001), extension angle (*r_s_*_ _= −0.39; *P *< 0.001), and relative load exposure (*r_s_*_ _= −0.34; *P *< 0.004). Echo-types distribution was beneficially associated with time from ACL injury (*r_s_* range: −0.20/ −0.32; *P *< 0.05).

**Discussion:**

ACL injured athletes displayed knee pain, extension deficit, and weaker quadriceps in the injured leg. While there were no differences in patellar tendon quality between legs, longer time from ACL injury showed better tendon quality.

## 1. Introduction

Anterior cruciate ligament (ACL) injuries are a significant cause of time loss in sport (1–3). Frequently, patients with ACL tear (ACLp) present with swelling, knee pain, and restricted range of motion (ROM) that might lead to a protective gait pattern (4). The location and intensity of pain varies depending on the severity of the associated lesions such as meniscal and collateral ligaments injuries (5).

One of the main functions of the intact ACL is to control the anterior translation of the tibia in the last degrees of knee extension (3, 6–8). Thus, it is not surprising that ACLp adopt compensations during gait to avoid this vulnerable position (4, 9–11). More specifically, increased flexion and reduced internal rotation with a resulting reduction in ROM (pivot-shift avoidance gait) have been observed in ACLp to improve stability at the late stance phase of walking (7, 9, 10). Alternatively, acute ACLp (up to 6 months post ACL injury) might keep the injured knee stiffer at an extended position (quadriceps avoidance pattern) to reduce the anterior tibial translation during walking (7, 11–13).

Biomechanical gait analysis of ACLp have demonstrated lower quadriceps and gastrocnemius activation, lower vertical ground reaction forces, and lower joint loads in the injured leg at weight acceptance in comparison to the uninjured and/or control leg (14). Interestingly, in acute ACLp, even a small quadriceps strength deficit was sufficient to cause altered weight-bearing in functional tasks such as stepping up and down (15).

Muscles and tendons are active structures with high capacity to adapt to different demands (16–20). Offloading the lower limb after an injury is a common compensatory mechanism that might reduce pain and improve function (14, 15). However, a short period of offloading the lower limb seemed to negatively affect the patellar tendon quality by progressively reducing the stiffness and the collagen synthesis rate (21–23). A normal tendon is characterized by the absence of hypoechoic areas and/ or increased thickness in ultrasound images (16). Even though abnormal imaging features have not been associated to the presence and severity of pain in the tendon (24, 25), an abnormal tendon image in ultrasound at pre-season has been linked to three to fivefold increase in risk of developing tendon symptoms during the season (18, 25).

One method of exploring tendon quality is ultrasound tissue characterization (UTC), which is a valid and reliable tool to assess healthy (26–28), pathological (26–28), and harvested tendons (29). The UTC grading system is based on histopathological studies that correlated the structural organization of equine tendon specimens with ultrasound images (30–33). It categorizes the quality of the tendon tissue from more to least organized (echo-types I–IV) (27).

UTC has been increasingly used to quantify load effects in patellar and Achilles' tendons. A reactive tendon response to load have been proposed by Cook's tendinopathy continuum model. In this phase, with appropriate load management, the tendon would return to non-reactive normal state (34). Conversely, it has been proposed that higher cumulative loads might alter tendon homeostasis resulting in swelling and/or increased waviness of the tendon bundle. These adaptations may occur within days of change in load exposure (35). Such adaptation was observed in UTC scans as reduction in the proportion of echo-type I with an increase of echo-type II, but no significant changes in the proportion of echo-types III and IV (disorganized tendon tissue) (36, 37).

It is important to consider the patellar tendon quality during the rehabilitation after an ACL injury, as it is one of the most common grafts used to replace a torn ACL (38, 39). It is unknown whether the uneven loads between ACLp injured and uninjured legs (14, 15) would offload the patellar tendon in a way that could cause acute adaptations within these tendons. Thus, quadriceps strength, patellar tendon quality, and the associated symptoms of perceived knee stability, relative load exposure, and extension angle in ACL injured athletes may have important implications for rehabilitation before and after the ligament reconstruction.

Consequently, the first aim of the study was to evaluate and compare the quadriceps strength, the patellar tendon quality, and the knee extension angle between injured and uninjured legs of athletes with a unilateral ACL tear participating in preoperative rehabilitation. The secondary aim was to find out if knee pain, extension angle, relative load exposure, and perceived knee stability are related to quadriceps strength, and consequently to patellar tendon quality. We hypothesized that the injured leg of ACLp will demonstrate limited extension range, lower quadriceps strength, smaller proportion of echo-type I, and greater proportion of echo-type II in the patellar tendon in comparison to the uninjured leg. We also hypothesized that the greater the extension deficit, the higher the knee pain, the lower the quadriceps strength, and patellar tendon quality.

## 2. Methods

### 2.1. Participants

A total of 412 male ACL injured athletes awaiting surgical reconstruction were examined in the assessment center of Aspetar, Orthopaedic and Sports Medicine Hospital from July 2015 to March 2020. Eighty-one athletes with age between 18 and 45 years, who had a unilateral ACL rupture confirmed by magnetic resonance imaging and had been referred to start or had enrolled in the ACL rehabilitation program of the same facility were selected to take part in the study. Patients were excluded if they did not match the age criteria (*n* = 15), presented with bilateral (*n* = 40) or previous ACL reconstruction in the contralateral leg (*n* = 263), declined to take part (*n* = 5), or presented inability to flex the affected knee sufficiently to acquire a proper image of the patellar tendon (*n* = 8) (37).

Informed consent was obtained from each participant. The study protocol meets the ethical standards in Sport and Exercise Science Research (40), and was approved by the ethical committee of the Anti-Doping Laboratory Qatar Research Office (2017000227).

### 2.2. Clinical assessment

Each participant's medical history and demographics were recorded, including age, height, current body mass, and date of injury. Participants were asked specifically about the presence and location of knee pain, including anterior, medial, lateral, posterior, and inside the knee pain. They were also asked about their perception of stability in the injured knee in relation to the uninjured stable knee. Values between 0 and 10 in the numeric rating scale (NRS) (41, 42) were used to obtain the subjective scores of maximum knee pain (NRS-knee pain), and perceived knee stability (NRS-stability) reported over the 7 days period prior to the assessment.

A digital inclinometer was used to quantify the passive knee extension angle (43). Negative values indicate hyperextension, while zero and positive values indicate deficit of extension.

### 2.3. Relative load exposure

Participant's gait and relative load exposure over the 7 days period prior to the assessment were considered. An ordinal classification was used to quantify levels of load exposure. These levels of load were 0 = no weight-bearing in the injured leg; 1 = participant was walking with partial weight-bearing while using 1 or 2 crutches but hadn't started the rehabilitation program; 2 = participant was walking with partial weight-bearing while using 1 or 2 crutches and had started no weight or partial weight-bearing exercises in rehabilitation; 3 = participant was walking with full weight-bearing but hadn't started rehabilitation; 4 = participant was walking with full weight-bearing and had started weight bearing exercises in rehabilitation; 5 = participant was walking with full weight-bearing and had started resisted exercises in rehabilitation; 6 = participant was performing resisted exercises and running in rehabilitation; 7 = participant was performing resisted and plyometrics exercises, and running in rehabilitation; 8 = participant was engaged in resisted exercises, plyometrics, running, and sports specific drills in rehabilitation.

### 2.4. Isokinetic strength test

A Biodex ^TM^ dynamometer (Biodex ^TM^, Shirley, New York) was used for the evaluation of quadriceps strength of each leg of ACLp. After warming up on a stationary bicycle for approximately 10 min, participants were seated upright with 90° of hip and knee flexion. After a brief familiarization, the participants were asked to perform five repetitions of maximal knee extension and flexion for each leg at 60°/s (3). Concentric peak torque for knee extension relative to body mass (Nm/kg) was recorded for the uninjured and injured legs of ACLp, in this order. Negative values of deficits indicate that the injured leg generated greater torque than the uninjured leg.

### 2.5. Ultrasound tissue characterization (UTC) acquisition, analysis, and processing

**Figure 1:**
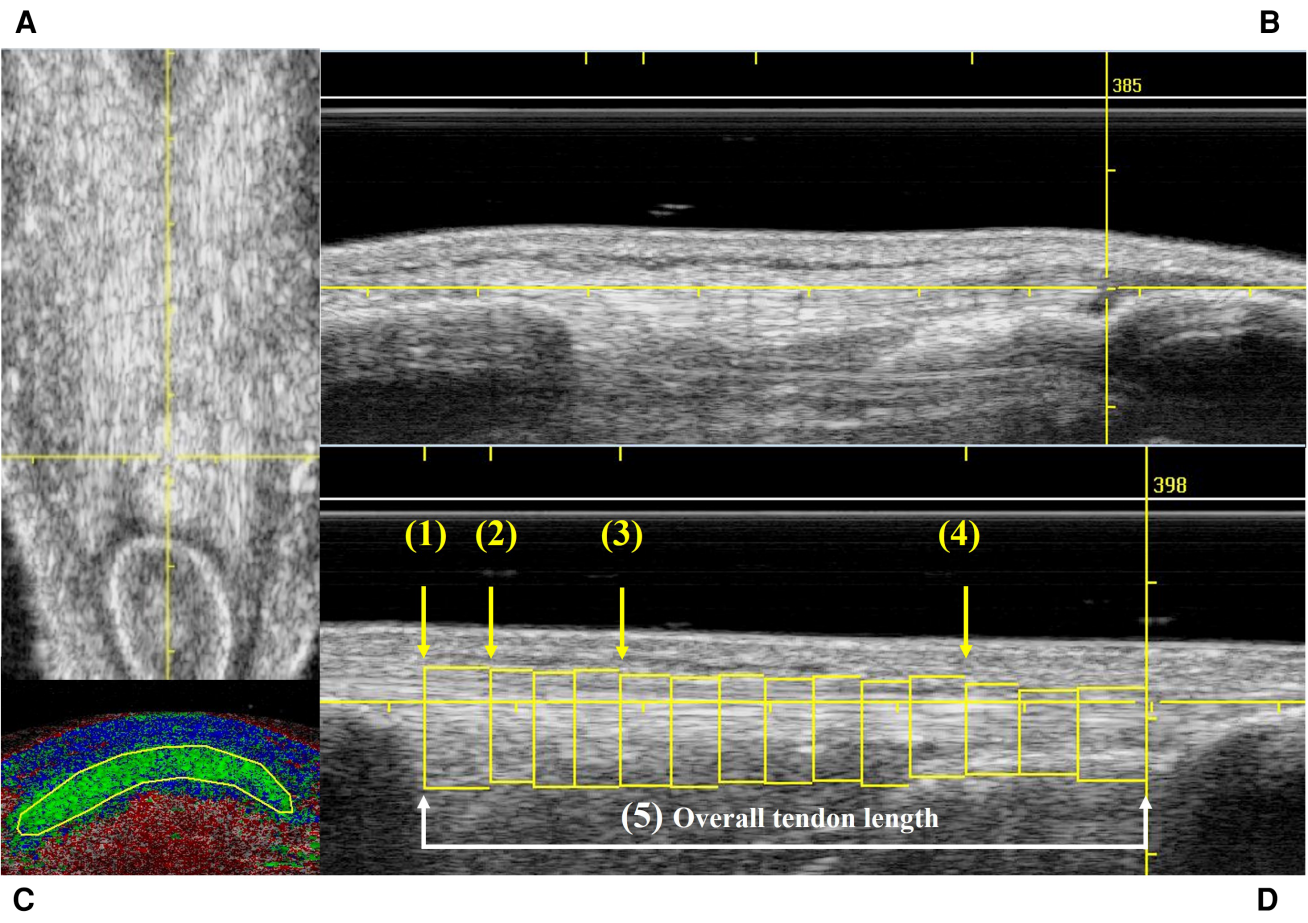
Example of UTC scans that met the inclusion criteria in frontal (**A**) and sagittal (**B**) views; contour delimitating the patellar tendon in transversal view (**C**); and the 5 studied areas of the patellar tendon (**D**). (**A**) Yellow reference line used to check the vertical alignment in the frontal view. (**B**) Yellow reference line used to check the horizontal alignment in the sagittal view. (**C**) Transverse view of the UTC color coded image showing a manually traced contour to delineate the area of the patellar tendon. (**D**) Sagittal view of the patellar tendon illustrating all the contours traced in the entire length of the patellar tendon. Yellow arrows show the four levels of the tendon (1–4) where the proportion of echo-types were calculated to characterize the tendon quality. (1) Patellar apex = first image after the patellar apex disappears in the transverse view. (2) Proximal tendon = at 0.5 cm from patellar apex. (3) Mid tendon = at 1.5 cm from patellar apex. (4) Distal tendon = at 75% of the tendon length. (5) White line and arrows show the distance between the 1st and last contours which characterizes the overall tendon length.

Both patellar tendons of the participants were scanned by a single experienced examiner (CSP) using UTC. This method uses a 5–12 MHz ultrasound transducer (SmartProbe 12L5, Terason 2000, Teratech, USA) Diagnostic Radiology (RRID:SCR_004427) fixed into a tracking device (UTC Tracker, UTC imaging, Netherlands). One UTC acquisition yields about 600 sequential transverse images in gray scale at regular intervals of 0.02 cm (26, 27). Each participant lay supine on the plinth with their knees flexed to approximately 100°. The UTC tracker was placed parallel to the long axis of the tendon resting with full contact on the anterior surface of the knee to acquire the patellar tendon images from proximal to distal. Ultrasound parameters were standardized as 12 MHz, with a focus at 2.8 cm and a depth of 4 cm (29). To minimize image artefacts and to ensure the ultrasound transducer is perpendicular to the tendon fibers, only scans that presented with the patellar apex and tibial tuberosity aligned longitudinally in the coronal view (Figure 1A), and a horizontal and taut patellar tendon in the sagittal view (Figure 1B) were included for analysis.

UTC analyses were performed on the UTC analyzer v.2.0.2 with window size 17. The UTC images in grey scale were inspected to identify areas of disorganization and/or increased thickness in the patellar tendons. Afterwards, in the transverse view of the UTC analyzer several contours were manually traced with a maximum of 0.5 cm apart (Figure 1C) along the patellar tendon length for each tendon (Figure 1D). This step defines the area where the UTC software, based on the stability of the echo-pattern, identifies the alignment of tendon fibers, and calculate the proportion of aligned and disorganized structures (%) within the tendon (26). To characterize the tendon quality, the UTC algorithm yield the proportion of echo-types I (green—aligned collagen bundles), II (blue—wavy collagen bundles), III (red—loose matrix), and IV (black—mainly amorphous matrix) in each area of interest (26, 31, 44). Echo-types I & II have been described as normal tendon or aligned fibrillar structure, while echo-types III & IV have been named as disorganized tissue structure (24).

The areas of interest in the patellar tendon were: (1) apex = the first image after the patellar apex disappears in the transverse view; (2) proximal tendon = 0.5 cm distal to the apex; (3) mid tendon = 1.5 cm distal to the apex; (4) distal tendon = at 75% of the tendon length; and (5) overall tendon, which includes all the contours drawn from the patellar apex to the tibial notch (Figure 1D). Additionally, the UTC algorithm also calculates the tendon volume (cm^3^) which is characterized by the area within a selected contour (patellar apex level).

A detailed protocol for the patellar tendon data acquisition, analysis of the intra- and inter-rater reliability as well as the minimal detectable changes (MDC) for the quality of the patellar tendons of ACLp athletes have been previously described (29).

### 2.6. Statistical analysis

Descriptive statistics including means, standard deviations (SD), minimum (min), and maximum (max) were calculated for all variables of interest and are presented where applicable.

Data distribution for normality was tested using the Shapiro–Wilk test. As extension angle, knee pain, knee stability, and all the variables of patellar tendon quality were not normally distributed, non-parametric Wilcoxon signed ranks test was used to compare tendon quality between injured and uninjured knees. However, quadriceps strength was normally distributed and analyzed with the parametric paired *t*-test.

Cohen's coefficient (*d*) was used to estimate the effect sizes (ES). Thresholds for small, medium, and large effect sizes were 0.2, 0.5, and 0.8, respectively (45).


d=(MeanACLp(injured)−MeanACLp(uninjured))/SDpooled

Spearman's rank-order correlation (*r_s_*) was used to assess the strength and direction of the association among the variables: quadriceps strength, proportion of echo-types in the patellar tendon, NRS-knee pain, NRS-stability, knee extension angle, relative load exposure, and time from ACL injury. *r_s_* values were ranked as: very weak or no correlation (when less than 0.19), weak (0.2–0.39), moderate (0.4–0.59), strong (0.6–079), or very strong (above 0.8) (46, 47).

Statistical significance was set as *P* < 0.05. SPSS v.28 was used for all statistical analyses (SPSS Inc., Chicago, Illinois, USA) SPSS (RRID:SCR_002865).

## 3. Results

### 3.1. Characteristics of the participants

The characteristics of the 81 ACLp at the time of data acquisition are detailed in Table 1. Of these athletes, 38 were footballers, 9 played handball, 6 basketball, 6 rugby, 5 futsal, 5 volleyball, 2 field hockey, and one in each of the following sports: athletics, beach soccer, billiards, cycling, rowing, sky diving, swimming, table tennis, tennis, and wrestling.

**Table 1:** Descriptive statistics of the characteristics of ACLp.

Characteristics	Mean ± SD (min-max)
Body mass (kg)	79.5 ± 14.5 (48.7–122.5)
Height (m)	1.78 ± 0.09 (1.55–2.03)
Age (years)	25 ± 5 (18–44)
Days from ACL injury (days)	61 ± 92 (6–622)
NRS—knee pain—injured knee (0–10)	3.1 ± 2.2 (0–8)
Reported stability (0–10)—Injured knee	6.3 ± 2.5 (0–10)

ACL, anterior cruciate ligament; ACLp, participants with a torn anterior cruciate ligament; SD, standard deviation; min, minimum; and max, maximum; NRS, numeric rating scale.

Their perceived stability in the injured knee ranged from 0 to 10 with average of 6.3 ± 2.5. Ten participants reported to feel the injured knee as stable as the uninjured (10/10). The passive extension angle ranged from −10° to 5° and from −10° to 2° in the injured and uninjured knees respectively. The difference in extension angle between injured and uninjured knees ranged from −5° to 10° with an average of 2.0 ± 2.7° of extension deficit. From the total sample, 41 participants displayed deficits of passive extension in the injured knee (≥0°), and from these, 38 complained of knee pain at the time of data acquisition.

Only one participant complained of anterior knee pain (NRS = 1/10) in the uninjured knee at the time of data acquisition. The average pain in the injured knee was 3.1 ± 2.2 (ES = 2.0; *P* < 0.001), however, 15/81 participants reported no pain in the injured knee.

None of the participants completely offloaded their injured leg (level 0), 1 participant was using crutches while adopting partial weight-bearing in the injured leg and was assessed before starting the rehabilitation program (level 1), 11 participants were using crutches in partial weight-bearing and were attending the rehabilitation program (level 2), 22 ACLp were in full weight-bearing and were assessed before starting the rehabilitation program (level 3), 16 ACLp were in full weight-bearing and were engaged in body weight exercises in rehabilitation (level 4), 29 participants were engaged in resisted exercises in rehabilitation (level 5), and 2 participants were engaged in resisted exercises, plyometrics, running, and sports specific drills in rehabilitation (level 8) at the time of data acquisition.

### 3.2. Visual inspection of the patellar tendons in grey scale images

The UTC images in grey scale revealed that 16 out of 81 ACLp displayed a clear disorganized area within the patellar tendon, namely, increased thickness and/ or hypoechogenic areas. Seven of these participants presented one or more of these features in both patellar tendons, 5/16 in the tendon of the injured leg and 4/16 in the uninjured leg. While only 2 of these tendons were associated with anterior knee pain specifically at the time of data acquisition, 5 out of these 16 ACLp had a prior history of unilateral tendinopathy. From the 16 athletes with abnormal features in the tendon, 8 played football, 3 basketball, 2 rugby, 1 volleyball, 1 handball, and 1 field hockey.

**Figure 2:**
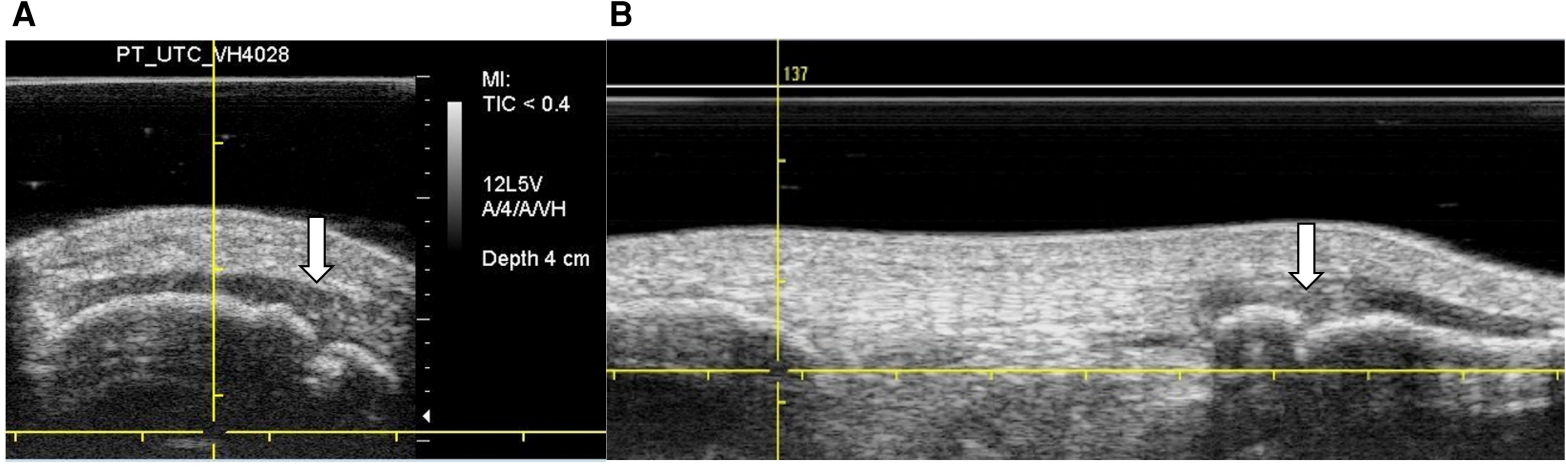
UTC images in grey scale—findings in the patella and tibial tuberosity. (**A**) Transverse view—white arrow shows case of Sinding–Larsen and Johansson sign in the patella. (**B**) Sagittal view—white arrow shows case of Osgood Schlatter sign at the tibial tuberosity.

The UTC scans also revealed that 3 ACLp displayed signs of Sinding-Larsen and Johansson syndrome (Figure 2A), whilst 5 ACLp showed signs of Osgood Schlatter disease (Figure 2B) in one of their tendons (48, 49).

### 3.3. Quadriceps strength

Eleven participants were unable to perform the isokinetic strength test due to concern of flaring up symptoms in the involved knee just before surgery. Comparisons between the injured and uninjured legs of ACLp revealed a significant reduction in quadriceps strength in the injured leg (ES = 1.2, *P* < 0.001) (Table 2). The difference in quadriceps strength between injured and uninjured legs was greater than the MDC previously calculated for the same variable in patients with knee osteoarthritis (50). Participants displayed on average 21.7% ± 19.3% deficit in quadriceps strength in relation to the uninjured leg, with values ranging from −35.5% to 72.1%. Only 8 out of 70 ACLp generated greater extension torque in the injured leg.

**Table 2:** Comparisons between the injured and uninjured legs of ACLp.

ACLp (*n* = 81)	Injured leg	Uninjured leg	Inj × UnInj	
	Mean ± SD	Mean ± SD	*p*-values	Effect size
Echo-type I (%)				
Patella apex^a^	55.5 ± 14.3	54.4 ± 17.0	0.59	0.07
Proximal tendon^a^	61.4 ± 12.5	63.3 ± 14.6	0.21	0.14
Mid tendon^a^	65.0 ± 12.5	66.8 ± 12.6	0.28	0.14
Distal tendon^a^	57.2 ± 15.3	55.5 ± 18.6	0.25	0.10
Echo-type II (%)				
Patella apex^a^	37.7 ± 11.4	36.6 ± 11.7	0.43	0.09
Proximal tendon^a^	34.4 ± 10.5	33.2 ± 12.5	0.31	0.10
Mid tendon^a^	31.3 ± 10.2	29.5 ± 10.5	0.20	0.17
Distal tendon^a^	34.0 ± 11.6	33.6 ± 11.6	0.95	0.03
Echo-type III (%)				
Patella apex^a^	5.0 ± 9.0	6.8 ± 12.0	0.22	0.17
Proximal tendon^a^	2.7 ± 5.4	2.3 ± 3.9	0.77	0.09
Mid tendon^a^	2.4 ± 4.6	2.4 ± 4.9	0.73	0
Distal tendon^a^	6.1 ± 8.5	8.2 ± 11.6	0.22	0.21
Echo-type IV (%)				
Patella apex^a^	1.9 ± 4.7	2.2 ± 3.7	0.45	0.07
Proximal tendon^a^	1.4 ± 3.7	1.1 ± 2.4	0.60	0.10
Mid tendon^a^	1.3 ± 4.3	1.1 ± 2.9	0.95	0.05
Distal tendon^a^	2.5 ± 4.4	2.7 ± 3.8	0.76	0.05
Overall tendon (%)				
Echo-type I^a^	59.3 ± 10.9	58.9 ± 11.5	0.67	0.04
Echo-type II^a^	32.7 ± 8.3	31.9 ± 7.9	0.46	0.10
Echo-type III^a^	5.7 ± 5.7	6.6 ± 6.4	0.16	0.15
Echo-type IV^a^	2.4 ± 3.9	2.6 ± 3.1	0.18	0.06
Echo-types I + II^a^	92.0 ± 9.3	90.8 ± 9.3	0.25	0.13
Echo-types III + IV^a^	8.0 ± 9.3	9.2 ± 9.3	0.19	0.13
Volume (cm^3^)				
Patella apex^b^	0.9 ± 0.2	0.9 ± 0.2	0.64	0
Proximal tendon^a^	0.9 ± 0.2	0.9 ± 0.2	0.45	0
Mid tendon^b^	0.9 ± 0.2	0.9 ± 0.2	0.49	0
Distal tendon^a^	0.9 ± 0.2	0.8 ± 0.2	0.94	0.5
Range of motion (°)				
Passive knee extension^a^	−0.7 ± 3.1 (−10–5)	−2.7 ± 2.2 (−10–2)	<0.001*	0.7
PT/BM (Nm/kg)^b^				
Maximal quadriceps (*n* = 70)	227.1 ± 65.1*	287.3 ± 54.5*	<0.001*	1.2

ACLp, participants with a unilateral anterior cruciate ligament tear; BM, body mass; Inj, injured leg; UnInj, uninjured leg; PT/BM, peak torque divided by body mass.

^a^ Wilcoxon signed ranks test.

^b^ Paired samples test.

* Statistical difference between injured and uninjured legs.

### 3.4. Patellar tendon quality

There was no significant difference in volume and in echo-types distribution in any of the studied areas of the patellar tendon (*P* > 0.05) (Table 2).

### 3.5. Associations between the variables of interest

When considering both legs of all ACLp included in the current study, there was a weak association between quadriceps strength and knee extension angle (*r_s_* = −0.39; *P* < 0.001), moderate associations between quadriceps strength and NRS-knee pain (*r_s_* = −0.47; *P* < 0.001), and between NRS-knee pain and knee extension angle (*r_s_* = 0.40; *P* < 0.001).

When considering only the injured leg, there were weak associations between: quadriceps strength and relative load exposure (*r_s_* = 0.34; *P* < 0.004), quadriceps strength and NRS-stability (*r_s_* = 0.39; *P* < 0.001), NRS-stability and knee extension angle (*r_s_* = −0.34; *P* < 0.002), NRS-stability and relative load exposure (*r_s_* = 0.36; *P* < 0.001), and relative load exposure and NRS-knee pain (*r_s_* = −0.27; *P* < 0.01).

Regarding the correlations between echo-types distribution (I to IV in the 5 areas of the tendon) in both legs of all ACLp, there were no meaningful associations between quadriceps strength, NRS-knee pain, or knee extension angle with the distribution of the four echo-types regardless of tendon area (Table 3). However, there were weak to moderate associations between time from ACL injury and echo-types I to IV at different tendon levels (Table 3). There was a statistically significant association between quadriceps strength and echo-type III at the proximal tendon (*r_s_* = −0.17; *P* < 0.03) but the correlation strength is considered very weak or none (Table 3).

**Table 3:** Values of the associations between studied variables.

	NRS-knee pain (*n* = 162)		Quadriceps strength (*n* = 140)		Time from ACL injury (*n* = 162)		Knee extension (*n* = 162)	
	*r_s_*	*p*-value	*r_s_*	*p*-value	*r_s_*	*p*-value	*r_s_*	*p*-value
NRS-knee pain	–	–	−0.47	<0.001*	0.02	0.83	0.40	<0.001*
Quadriceps strength	−0.47	<0.001*	–	–	0.05	0.55	−0.39	<0.001*
Time from ACL injury	0.02	0.83	0.05	0.55	–	–	−0.01	0.90
Echo-type I								
Patella apex	−0.05	0.51	0.08	0.35	−0.06	0.41	−0.12	0.12
Proximal tendon	−0.07	0.36	0.14	0.09	−0.03	0.69	−0.07	0.34
Mid tendon	0.01	0.93	0.05	0.59	0.09	0.25	−0.01	0.88
Distal tendon	0.09	0.21	−0.04	0.63	0.30	<0.01*	−0.03	0.63
Echo-type II								
Patella apex	0.09	0.22	−0.11	0.18	0.25	<0.01*	0.13	0.10
Proximal tendon	0.07	0.35	−0.18	0.03*	0.20	0.01*	0.07	0.37
Mid tendon	0.01	0.90	−0.12	0.17	0.09	0.23	−0.04	0.61
Distal tendon	−0.01	0.92	0.01	0.90	0.08	0.27	0.03	0.70
Echo-type III								
Patella apex	−0.01	0.95	−0.09	0.28	−0.21	<0.01*	0.09	0.26
Proximal tendon	−0.04	0.63	−0.09	0.27	−0.35	<0.01*	0.03	0.76
Mid tendon	−0.04	0.61	−0.11	0.21	−0.33	<0.01*	0.09	0.26
Distal tendon	−0.06	0.43	−0.03	0.70	−0.34	<0.01*	0.01	0.90
Echo-type IV								
Patella apex	−0.02	0.79	−0.08	0.32	−0.28	<0.01*	0.06	0.49
Proximal tendon	−0.06	0.41	−0.03	0.71	−0.41	<0.01*	−0.01	0.93
Mid tendon	−0.05	0.49	−0.04	0.64	−0.35	<0.01*	0.09	0.24
Distal tendon	−0.04	0.57	−0.05	0.55	−0.35	<0.01*	0.00	0.97

Variables of tendon quality, echo-type distribution in the specific areas of interest; NRS-knee pain, numerical rating scale for knee pain at the time of data acquisition; Quadriceps strength, maximal knee extensor's peak torque divided by body mass; Time from ACL injury, number of days between ACL injury and the time of data acquisition; Knee extension, angle of passive knee extension measured in the assessment; *r_s_*, Spearman correlation coefficient.

* Statistically significant.

**Figure 3:**
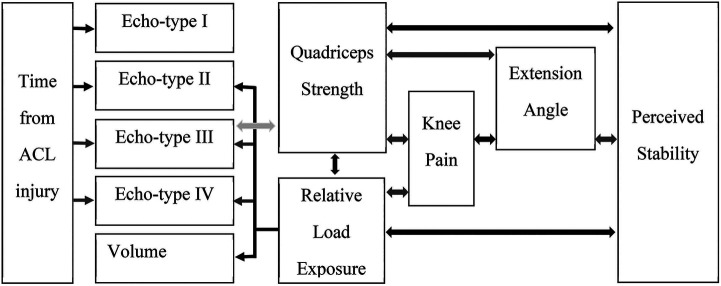
Summary of the associations among the studied variables. Black arrows = positive correlation with weak to moderate statistical significance. Dark grey arrows = negative correlation with weak to moderate statistical significance. Light grey arrow = very weak to no correlation although statistically significant.

When we considered the patellar tendon quality in the injured leg only, there were weak associations between relative load exposure and echo-type II at distal tendon (*r_s_* = 0.22; *P* < 0.04), echo-type III at patellar apex (*r_s_ *= −0.25; *P* < 0.02) and proximal tendon (*r_s_* = −0.24; *P* < 0.04), echo-type IV at patellar apex (*r_s_* = −0.26; *P* < 0.02), and volume at distal tendon (*r_s_* = 0.23; *P* < 0.04). Figure 3 depicts a representation of all the associations among the studied variables.

## 4. Discussion

To our knowledge, this is the first study to explore quadriceps strength, patellar tendon quality, reported knee stability, knee extension angle, knee pain, and relative load exposure simultaneously in athletes before the reconstructive surgery for a unilateral ACL tear. The study is also novel in that it is the first one to use UTC to evaluate the offloading effects in tendons, and one of the few studies using UTC to evaluate the quality in the entire tendon length.

Our main findings showed that ACLp had significantly less knee extension, feeling of reduced stability, more knee pain, and reduced quadriceps strength in their injured compared to uninjured legs. These results agree with our initial hypothesis and with the extensive evidence describing the negative impact of an ACL injury in the extensor mechanism of the knee (51–56).

About 95% of the participants in the current study were less than 6 months from their ACL injury, so it was not surprising that more than 80% of ACLp reported pain in the knee and 50% were unable to fully extend their knees. Filbay et al., also noted that 57% of their participants with acute ACL rupture presented deficit of extension (57). We observed in the injured leg of ACLp an average of extension deficit of 2.0 ± 2.7°. Comparatively, Muneta et al., found an average of 1.3 ± 3.9° of extension loss but their 81 patients with ACL rupture had average of 612 days from injury (58). Likely, the presence of swelling in the knee might had contributed to these findings of extension loss. Although not considered in the present study, swelling is frequently present in ACL injured knees. It distends the joint capsule (59–61), restricts the range of motion, and may or may not cause pain (61, 62). In addition, even a mild swelling in a healthy knee was found sufficient to trigger quadriceps inhibition (63). Furthermore, more than half of ACLp in the current study were adopting partial weight-bearing or had only recently started to fully load their injured leg at the time of the assessment. It is possible that these compensations might have contributed to the significant smaller quadriceps strength observed in their injured legs (227.1 ± 65.1 Nm/kg) in comparison to the uninjured one (287.3 ± 54.5 Nm/kg). Acute ACL patients frequently adopt different strategies during gait to compensate the stability loss (4, 7, 9–11, 64–66). These patients tend to avoid using the quadriceps muscle close to knee extension to minimize the strain in the torn ligament (7). Compensatory mechanisms such as pivot-shift avoidance gait (13) or quadriceps avoidance pattern (7, 11, 12) directly affects the extensor mechanism of the knee by reducing the demand in the quadriceps muscle preventing excessive anterior tibial translation (7). Yet, regardless of the reason behind the quadriceps weakness, even a small deficit in quadriceps strength in the ACL injured leg was found sufficient to affect weight-bearing, increase the impact forces in both legs, and negatively impact function (15).

To the best of our knowledge, few studies have explored quadriceps isokinetic strength in ACL injured patients so soon after injury. Our participants had an average of 61 ± 92 days from the ACL injury at the time of assessment, and the injured leg presented average deficit of 21.7% ± 19.3% in relation to uninjured leg. However, not only the time since ACL injury poses a challenge when comparing strength values between studies. Gender, age, level of physical activity, test protocols, and the type of strength variable studied are additional factors hindering comparisons. For instance, de Jong et al., noted 17% of deficit in the injured leg after assessing the quadriceps strength in 191 (29 women) patients before the ACL reconstruction, but their average time from injury to surgery was 2.2 years pos- injury, and the exact time of the preoperative strength test was not specified (67). Thus, caution is advised when comparing the quadriceps peak torque in relation to body mass of our 81 male registered athletes (227.1 ± 65.1 Nm/kg) with that of the 36 (18 female) participants with 3.8 years from injury and a quadriceps peak torque of 174.9 ± 63.8 Nm (68).

Moreover, our findings indicated that quadriceps strength was weakly to moderately associated to knee pain, knee stability, knee extension, and relative load exposure, i.e., higher knee pain, greater deficit of extension, lower stability, and lower load in the lower legs, was associated to weaker quadriceps (Figure 3). Persistent quadriceps weakness has been previously linked to deficit of extension and anterior knee pain (52, 56, 69–71). Initially, it seems obvious that these symptoms would occur in parallel after such an incapacitating injury such as an ACL tear, however, each ACL patient might display each of these symptoms in very particular ways demanding personalized targeted interventions during rehabilitation. For instance, by exploring the data of relative load exposure in which ACLp were engaged, it was interesting to note how participants responded differently to the ACL injury, displaying different coping mechanisms and ability to tolerate the lack of ACL. Whilst we observed the majority of ACLp presenting with instability, protective behavior, pain, deficit of extension, and quadriceps weakness, we also witnessed that 9 participants (time from ACL injury ranging from 12 to 88 days) perceived no instability in their injured knee, had full range of motion, and no strength deficit. This minority of ACLp might be characterized as copers (72). It is noted that 5 of them opted for non-surgical treatment.

There is little evidence on the psychological impact of persistent symptoms after an ACL injury (73–75). Most of the studies focus on psychological readiness to return to sports (76–78). For Chmielewski et al., preoperative psychosocial status did not reflect pain and function at 3 months post ACL reconstruction (74). However, increase in self-efficacy, optimism, and high motivation have been associated to positive functional outcomes (74, 76, 79). On the other hand, persistent pain, fear of movement, and anxiety are associated with a lower rate of return to sports (73, 76). Thus, the worsening or improvement in the parameters investigated in this study might impact these patients functionally and psychosocially interfering with their recovery process, readiness for surgery, postoperative rehabilitation, and return to sports (73).

Considering ACLp presented significant quadriceps deficiency in the injured leg and most of them adopted compensatory mechanisms during gait to offload the injured knee, it was unexpected to find that the patellar tendons in their injured and uninjured knees presented similar distribution of echo-types and volume. It was also unexpected that there was no meaningful association between quadriceps strength and tendon quality, even though the patellar tendon is a direct part of the extensor mechanism of the knee and responsible for transferring concentric and eccentric forces from the quadriceps to the tibia (16, 23, 80). The lack of association was especially surprising because offloading the lower leg of healthy individuals for a brief period, such as 1–3 weeks, reduced significantly the knee extensor torque, the muscle cross-sectional area, the synthesis of collagen rate within the tendons (21, 22), and increased the patellar tendon elongation along with the decrease in tendon stiffness (22, 23, 81).

Our initial hypothesis of tendon adaptations post-ACL injury was mostly rejected, however, in agreement with our findings of tendon volume, previous studies have reported that regardless of the period of rest and/or immobilization the tendon cross-sectional area remains the same (22, 82). On the other hand, many animal and human studies have reported the negative effects of offloading and/or immobilization on the mechanical properties of tendons (16, 21, 22, 83–86). It has been suggested that a period of disuse could cause tendon deterioration and a transient adaptation in echo-type distribution (36, 87) which did not happen in the patellar tendons of ACLp. It is possible though, that the interruption in sports and the offloading effect caused by the ACL injury led to a transient adaptation in both patellar tendons of ACLp, thus the lack of differences in patellar tendon quality between injured and uninjured knees. Or yet, that the compensations adopted by ACLp were not sufficient to trigger physical changes in the tendon fibers that would be visible in UTC scans. The latter seems plausible taking into account the echo-types distribution in both patellar tendons of ACLp is comparable with previous values described in the literature for the proximal portion of healthy (approximately 68%, 32%, nearly 1%, and 0%, for echo-types I, II, III and IV respectively), and asymptomatic (approximately 60%, 37%, 2%, 1%, for echo-types I, II, III and IV respectively) patellar tendons (37).

Another point to consider is that 58 ACLp were already engaged in rehabilitation for at least 7 days, which could have positively affected the tendons in their injured leg. Particularly when considering that some variables of patellar tendon quality were weakly to moderately associated to relative load exposure and time from ACL injury. These relationships suggest that tendon quality improved with increased loading and longer time from ACL injury, i.e., an increase in relative load was associated to an increase in the proportion of echo-types II, reduction of echo-types III and IV, and greater volume at the distal tendon. Whereas longer time from injury was associated to increase in echo-types I and II, and reduction of echo-types III and IV. In UTC scans, echo-types I and II are described as aligned fibrillar structure, while echo-types III and IV are described as disorganized tissue that lacks structural organization (26). Participants in the current study started body weight exercises followed by resisted exercises as soon as they were walking with full weight-bearing and the symptoms in the injured knee were adequately controlled. It appears that resistance training prevents the detrimental effects of bed rest in tendons (88). Also, there is some evidence suggesting that targeted loading improves the tolerance of the tendon, resulting in more aligned tendon tissue (89).

Furthermore, we observed a lack of significant associations between scores of knee pain with all the tendon variables studied. Although 16 participants displayed abnormal signs in their tendons, only 5 of them had history of tendinopathy, and only 2 out of 16 were symptomatic at the time of data acquisition (anterior knee pain). The presence of tendon abnormalities in UTC scans have not been associated to current symptoms in tendons (24, 90). However, a recent study using UTC proposed that an increased proportion of disorganized tendon structure (echo-types III + IV) in the Achilles (>8.5%) and patellar tendons (>10%) at baseline have been associated with increased risk of developing lower leg pathologies during periods of increased training (91). These findings suggest that investigating the tendon quality before starting a rehabilitation/training program and identifying the participants with higher risk could lead to preventive strategies, with targeted interventions that might avoid flaring tendon symptoms and/or reducing injury occurrence.

Our findings are significant because they shed light on the impact of an ACL injury on muscle strength and tendon quality before the ligament reconstructive surgery. Since patellar tendons are often used as ACL graft, it is relevant to know the quality of the patellar tendons and its relations with quadriceps strength, load exposure, and knee pain in individuals waiting for the ACL reconstruction. At the time of data acquisition about half of ACLp presented deficit of extension, 66 of them had knee pain, and quadriceps strength deficit ranging from −35% to 75% of the uninjured leg. Further examination revealed that participants with extension deficits showed significantly greater deficits of quadriceps strength highlighting the importance of achieving full extension motion to improve quadriceps strength. Ideally, before the reconstructive surgery the injured knee would present minimal strength deficit in comparison to the uninjured leg, full knee extension, and no pain. Preoperative quadriceps strength deficits greater than 20% in comparison to the uninjured leg are often correlated to long lasting strength deficits after the ACL reconstruction (70). Concurrently, less anterior knee pain before the ACL reconstruction is predictive of better clinical outcomes at 12 months post-surgery (92, 93). Without a structured strengthening program, side-to-side deficits in quadriceps strength appeared to persist even 1.5 year after the ACL injury (54). Thus, structured rehabilitation prior to the ACL reconstruction has been advocated to restore knee function and reduce strength deficits (54, 94).

Even though all participants included in the study were active patients at the same institution and were enrolled in the same preoperative rehabilitation program, a few limitations need to be considered as they were not controlled for: the presence of associated knee injuries, the amount of swelling in comparison to uninjured knee, and the level of knee function. Due to our study group consisting of male registered athletes from 18 to 44 years of age, caution should be exercised when extrapolating these results for other age groups and female athletes. Additionally, to avoid misleading comparisons with other research, it is important to highlight that we used “window 17” in the UTC imaging software to analyze the quality of the patellar tendons of ACLp in the current study. It is likely that other (wider, narrower) window settings result in different output in the same tendons (24, 28, 29, 37).

In summary, feeling of instability, deficit of knee extension, pain in the knee, and deficit of quadriceps strength were common symptoms in athletes with ACL injury. There is an intricate relationship of reported knee stability and knee pain with deficits in knee extension, relative load exposure, and quadriceps strength that might dictate the pace of progression in rehabilitation.

The quality of patellar tendon improves with the increase in lower leg load and the time from ACL injury. And the lack of differences in patellar tendon echo-type distribution between the injured and uninjured legs of athletes sustaining a unilateral ACL injury suggests that the tendon of the uninjured leg can be used as a reference for future longitudinal studies.

Based on our results a clinician might assume, when treating ACL injured patients before the reconstructive surgery, that the ACL injury itself, the presence of knee pain, the reduced extension range, and the reduced quadriceps strength will not significantly affect the quality of their patellar tendons.

## Data Availability

The raw data supporting the conclusions of this article will be made available by the authors, without undue reservation.
